# Conjunctival sarcoidosis presenting as limbal conjunctival hypertrophy: a case report

**DOI:** 10.1186/1752-1947-8-63

**Published:** 2014-02-20

**Authors:** Sang Beom Han, Hee Kyung Yang, Joon Young Hyon, Won Ryang Wee

**Affiliations:** 1 Department of Ophthalmology, Kangwon National University Graduate School of Medicine, Kangwon National University Hospital, Chuncheon, Korea; 2 Department of Ophthalmology, Seoul National University College of Medicine, Seoul National University Bundang Hospital, 300 Gumi-dong, Bundang-gu, Seongnam, Gyeonggi 463-707, Korea; 3 Department of Ophthalmology, Seoul National University College of Medicine, Seoul National University Hospital, Seoul, Korea

**Keywords:** Conjunctival hypertrophy, Conjunctival sarcoidosis, Cyclosporine, Limbal hypertrophy, Sarcoidosis

## Abstract

**Introduction:**

To the best of our knowledge, this is the first report of a case of conjunctival sarcoidosis that presented as limbal conjunctival hypertrophy and was controlled with topical cyclosporine.

**Case presentation:**

A 70-year-old Asian woman presented with painless limbal conjunctival hypertrophy. An incisional biopsy of the hypertrophic lesion showed noncaseating granulomas that consisted of multinucleated giant cells and epithelioid cells. The results of a systemic evaluation were normal except for a slight increase in serum angiotensin-converting enzyme to 58.9IU/L (normal range, 8 to 55IU/L). Twice-daily 0.05% topical cyclosporine was prescribed for the treatment of remaining lesions. After two months, the lesions had completely resolved and her serum angiotensin-converting enzyme level had normalized.

**Conclusions:**

Sarcoidosis should be considered in the differential diagnosis of conjunctival hypertrophy. Topical cyclosporine can be useful in conjunctival sarcoidosis.

## Introduction

Sarcoidosis is a multisystemic inflammatory process characterized by noncaseating granulomatous infiltration [1]. Ocular involvement is found in 20% to 50% of patients; the most common complaint (30% to 70%) is uveitis caused by an accumulation of T cells and mononuclear cells [1,2]. Conjunctival involvement is also frequently encountered (40%) and usually manifests as conjunctival nodules associated with the formation of noncaseating granulomas [1-3]. Although corticosteroids have been the mainstay of treatment in ocular sarcoidosis, chronic use is frequently associated with various side effects [3]. Thus, steroid-sparing agents, including minocycline and cyclosporine, have been introduced for the treatment of ocular sarcoidosis [2-4].

We present the case of a patient with isolated conjunctival sarcoidosis presenting as limbal conjunctival hypertrophy who was successfully treated with 0.05% topical cyclosporine.

## Case presentation

**Figure 1:**
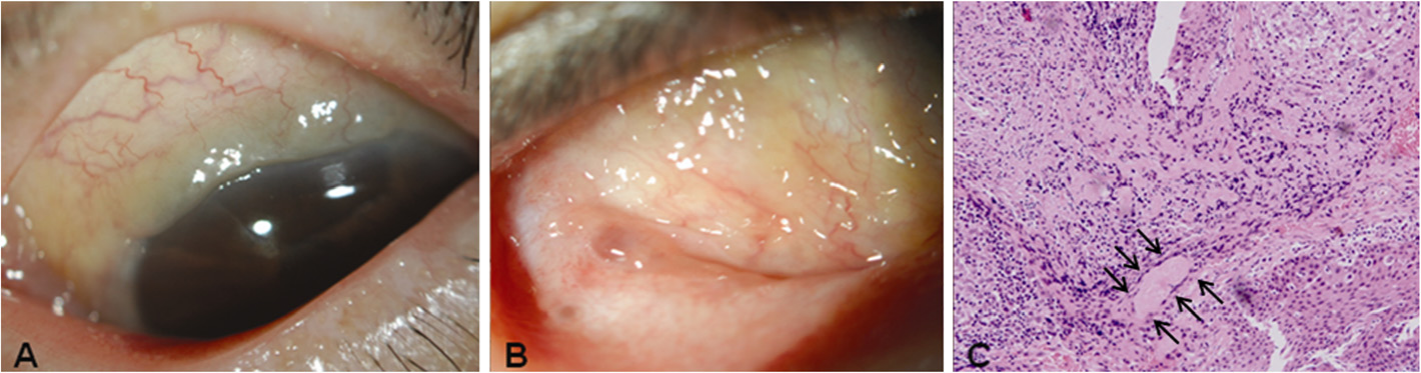
**Anterior segment photography and histologic examination at presentation. (A)** Anterior segment photography of the left eye at presentation. Yellowish conjunctival hypertrophy around the limbus is observed. **(B)** Anterior segment photography of the right eye at presentation. Multiple yellowish conjunctival nodules are observed. **(C)** Histological examination shows noncaseating granulomas that consist of multinucleated giant cells and epithelioid macrophages (black arrows) in the conjunctival stroma (hematoxylin and eosin staining, original magnification × 200).

**Figure 2:**
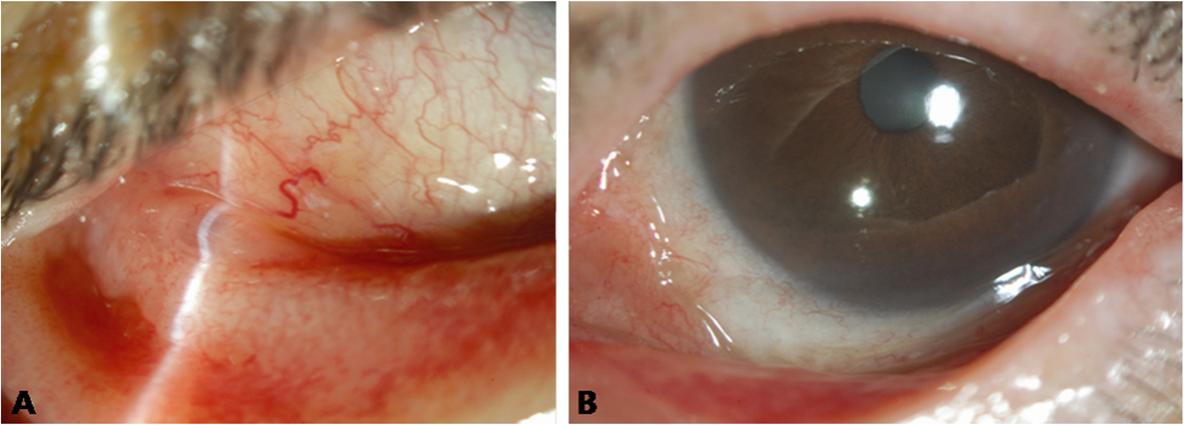
**Anterior segment photography after treatment. (A)** Anterior segment photography of the right eye taken after treatment with topical cyclosporine. Complete resolution of conjunctival nodules is observed. **(B)** Anterior segment photography of the left eye taken after treatment with topical cyclosporine. Resolution of the limbal conjunctival hypertrophy is observed. The superior part of the hypertrophic conjunctiva was removed with incisional biopsy.

A 70-year-old Asian woman was referred to our hospital for an evaluation of limbal conjunctival hypertrophy in her left eye. She reported mild discomfort but had no other symptoms, such as pain or itching. She had chronic angle closure glaucoma in her left eye and was on 0.1% brimonidine twice a day and 0.005% latanoprost four times a day. Her past medical history was unremarkable except for hypertension and hyperlipidemia. A slit-lamp examination showed multiple yellowish conjunctival nodules in both eyes and hypertrophy around the superior limbal area in her left eye (Figure 1A,B). An incisional biopsy of the superior conjunctival tissue revealed noncaseating granulomas that consisted of multinucleated giant cells and epithelioid cells (Figure 1C). No evidence of fungi, acid-fast bacilli or foreign bodies was found. A systemic evaluation, including a physical examination, chest X-ray and high-resolution computed tomography, pulmonary function tests, and an analysis of serum angiotensin-converting enzyme (ACE), antineutrophil cytoplasmic antibody, and serum and urinary calcium were performed. All results were normal except a mildly increased serum ACE level of 58.9IU/L (normal range, 8 to 55IU/L). She was prescribed 0.05% topical cyclosporine twice daily for the treatment of the remaining conjunctival nodules. Two months later, the nodules had completely resolved (Figure 2), and her serum ACE level had normalized to 44.1IU/L. The topical cyclosporine was discontinued.

## Discussion

Although conjunctival sarcoidosis mostly presents as multiple nodules resembling follicular conjunctivitis, other conjunctival manifestations including conjunctival deposit, large conjunctival tumor and multilobular limbal nodules have been reported [1-3,5-7]. The differential diagnosis of limbal hypertrophy includes vernal keratoconjunctivitis, atopic keratoconjunctivitis, allergic conjunctivitis, superior limbic keratoconjunctivitis, trachoma, ligneous conjunctivitis, soft contact lens complication, and conjunctival tumors such as papilloma, lymphoma and intraepithelial neoplasia [8,9]. However, sarcoidosis has rarely been considered in the differential diagnosis, and in fact, our patient was referred to us with an impression of conjunctival neoplasia. In most cases of conjunctival sarcoidosis, the granulomatous inflammation results in conjunctival nodules. However, in our patient, there was a large amount of granulomatous infiltration around the superior perilimbal area, and it may have led to the limbal conjunctival hypertrophy.

Cyclosporine is known to be useful in various ocular surface inflammatory diseases, such as ocular rosacea, dry eye disease, atopic keratoconjunctivitis, graft-versus-host disease and herpetic stromal keratitis, due to its anti-inflammatory and immunomodulatory action [10]. Although the pathogenesis of sarcoidosis is still unclear, cluster of differentiation (CD) 4 T cells appear to play an important role in granuloma formation [1,2]. Accordingly, cyclosporine is expected to be useful for its selective inhibitory effect on CD4+ T lymphocyte proliferation via inhibition of interleukin-2 receptor expression [1,3]. Oral cyclosporine is shown to be effective in vision-threatening ocular sarcoidosis as well as refractory systemic sarcoidosis [3]. Topical cyclosporine was also effective in the treatment of conjunctival sarcoidosis in steroid-responders [2,3]. We also chose topical cyclosporine instead of steroids due to pre-existing glaucoma in our patient. Unlike previous cases [2,3], our patient’s serum ACE level was elevated, and normalized in response to the cyclosporine treatment. In patients with sarcoidosis, serum ACE levels are elevated due to marked synthesis by epithelioid cells, thus the ACE level reflects the mass of ACE-producing granuloma cells [11]. Therefore, although an elevated serum ACE level is not a specific finding for sarcoidosis, serial measurements of serum ACE level are useful in the evaluation of treatment response. In our patient, her elevated ACE level normalized with the disappearance of conjunctival lesions, which suggests suppression of disease activity with the use of topical cyclosporine.

## Conclusions

Our experience suggests that sarcoidosis can manifest as limbal conjunctival hypertrophy, and should therefore be included in the differential diagnosis of sarcoidosis. Topical cyclosporine can be a useful option in the treatment of conjunctival sarcoidosis, especially when there are concerns about steroid side effects.

## Consent

Written informed consent was obtained from the patient for publication of this manuscript and accompanying images. A copy of the written consent is available for review by the Editor-in-Chief of this journal.

## Abbreviations

ACE: angiotensin-converting enzyme; CD: cluster of differentiation.

## Competing interests

The authors declare that they have no competing interests.

## Authors’ contribution

SBH and HKY acquired, analyzed and interpreted the patient data regarding the sarcoidosis. JYH and WRW performed the histological examination of the conjunctival tissue sample. SBH and HKY were major contributors in writing the manuscript, and JYH and WRW revised it critically. All authors read and approved the final manuscript.

## Authors’ information

SBH, JYH and WRW work in the corneal and refractive surgery division at Seoul National University College of Medicine, and HKY works in the strabismus and pediatric ophthalmology division at Seoul National University College of Medicine.
